# Coalition building by drug user and sex worker community-based organizations in Vietnam can lead to improved interactions with government agencies: a qualitative study

**DOI:** 10.1186/s12954-015-0070-1

**Published:** 2015-10-16

**Authors:** Leah T. Le, Lauretta E. Grau, Huong H. Nguyen, Oanh Hai T. Khuat, Robert Heimer

**Affiliations:** Yale School of Medicine, New Haven, CT 06511 USA; Center for Supporting Community Development Initiatives (SCDI), 240 Mai Anh Tuan, Ba Dinh district, Hanoi, Vietnam; Yale School of Public Health, New Haven, CT 06510 USA

**Keywords:** Vulnerable communities, Methadone maintenance treatment programs, Government recognition, Organizational sustainability

## Abstract

**Background:**

Drug users and female sex workers are among the groups most vulnerable to HIV infection in Vietnam. To address the HIV epidemic within these communities, former drug users and sex workers established the first community-based organizations (CBOs) in 2009. The study provides a focused assessment of CBOs’ expanding efforts to advocate for their members that identifies existing collaborations with Vietnamese government programs. This assessment explores the barriers to and facilitators of expansion in order to propose recommendations to improve the working relationship between CBOs and government programs.

**Methods:**

Thirty-two individuals from drug user and sex worker CBOs (*n* = 24) and relevant government programs (*n* = 8) participated in face-to-face interviews in Hanoi, Ho Chi Minh City, and Hai Phong. Coded interview transcripts were analyzed qualitatively concerning the purpose of CBOs, the interactions between CBOs and government programs, and the perceived barriers, facilitators, and feasibility of future CBO-government program collaborations.

**Results:**

Services provided by the CBOs were considered to improve members’ quality of life. The formation of coalitions among CBOs increased efficiency in meeting members’ specific service needs, in addition to internal capacity building. Government field staff interacted with CBOs by providing CBOs with technical and legal support. CBOs and methadone maintenance treatment (MMT) clinics collaborated to help the clinics meet patient enrollment quotas and facilitate entry into treatment for CBO members. Barriers to CBO-government program collaboration included perceived conflicting missions on how to address drug use and sex work in the community, limited CBO-government program communication, CBO mistrust of the MMT system, and lack of legal status for CBOs.

**Conclusion:**

To reduce these barriers, we recommend (1) introduction of CBO consultative services at government healthcare centers, (2) enlistment of CBO outreach to ensure full access to the imminent scaled-up MMT program, and (3) establishment of standards by which CBOs can obtain legal status.

## Background and rationale

### Current status of HIV in Vietnam

The first reported case of HIV in Vietnam occurred in 1990; the annual rate of HIV case ascertainment from 2009 to 2013 has been approximately 14,000 per year, and as of 2013, there were 254,000 people living with HIV/AIDS countrywide [1, 2]. Although this figure is relatively low, given a total population of nearly 90 million, it is consistent with a concentrated epidemic in which prevalence is high among people who inject drugs (PWID) and female sex workers [1]. Vietnam resembles several neighboring countries—Indonesia, Malaysia, Thailand, and the Philippines, for instance—in harboring a concentrated epidemic [3].

HIV prevalence among Vietnamese PWID in 2013 has been estimated at 10.3 % [1]. The provinces of Dien Bien and Quang Ninh have the highest HIV prevalence among PWID (56 %), followed by Hai Phong (48 %), Ho Chi Minh City (30 %), and Hanoi (25 %) [4, 5].

Female commercial sex workers are also at high risk for HIV infection. The HIV prevalence among sex workers in Hanoi, Ho Chi Minh City, and Hai Phong was approximately 22.5 %, 26.8 %, and 29.8 %, respectively [6–8]. Among 2986 sex workers participating in a survey in 12 provinces, 2.7 % reported a history of injecting drugs, of whom 30 % reported living with HIV/AIDS [9]. In 2013, the national HIV average prevalence among sex workers was 2.6 %, ranging from 0.3 % among venue-based sex workers to 23 % among street-based sex workers in part because street-based sex workers were more likely to inject drugs than were venue-based sex workers [1].

### Community-based organizations

Community-based grassroots organizations (CBOs) are defined as “public or private non-profit [organizations]…representative of a community or a significant segment of a community, and…engaged in meeting human, educational, environmental, or public safety community needs” [10]. Since the early 1980s, the harm reduction movement, often initiated by drug users themselves, has created CBOs in many parts of the world. Starting with the Amsterdam-based Interest Association for Drug Users in 1984 [11], other examples of autonomous organizations have emerged in Thailand, the USA, Denmark, the Netherlands, Australia, Brazil, Germany, and Russia [12, 13].

To address the concentrated HIV epidemic within these vulnerable groups, former drug users and sex workers began to establish CBOs in 2009. Consistent with the tradition of drug users actively participating in the harm reduction movement, CBOs in Vietnam typically are initiated, governed, and managed by their own community members, in this instance drug users or sex workers. Coordinating boards of the CBOs generally consist of the founders and the most respected and capable members of their community, whom we term peer educators. CBO activities are carried out by peer educators and additional outreach workers, some of whom may continue to be active drug users and sex workers.

The primary work of peer educators is to train outreach workers and interact with active drug users and sex workers in the community and in government detention centers, referred to as 05 (for sex workers) and 06 (for drug users) centers. These are managed by the Department of Social Evils Prevention (DSEP) within the Ministry of Labor, War Invalids, and Social Affairs (MoLISA) [14]. Since 2012, with the approval of the new Law on Sanction of Administrative Violations, sex workers are no longer sent to the 05 centers [15]. Work in both community and detention settings has led CBOs and their staff to engage with multiple branches of the Vietnamese government, including MoLISA, the Ministry of Health, and the police and judiciary systems. Assistance in navigating these systems has been provided to some CBOs in Vietnam through support from non-government organizations such as the Center for Supporting Community Development Initiatives (SCDI), the Global Fund to Fight AIDS, Tuberculosis, and Malaria, and the Hanoi and Ho Chi Minh City HIV/AIDS Associations. These non-governmental organizations collaborate with and provide material and technical support to CBOs and serve as brokers between the CBOs and donors or Vietnamese government programs. However, as CBOs grow, diversify, and take on new challenges, it is important to assess their needs as they seek to be civil society partners who are viewed by government officials as equals. One purpose of this study is to identify the challenges and suggest ways to improve the relationship between the CBOs and government agencies.

A second purpose is to consider the impact of economic changes. Although Vietnam has gained the economic status of a middle-income country, the change in status is being felt at the community level [16]. For example, funding for the CBO development, operations, and outreach activities, to date, has originated solely from international donors and, due to Vietnam’s newly attained economic status, foreign donors have begun to withdraw funding [1]. The Global Fund to Fight AIDS, Tuberculosis, and Malaria has implemented new priorities and allocation methods that will reduce funding to countries such as Vietnam in which the HIV epidemic is concentrated in high-risk populations [17]. Therefore, the CBOs must find other means of financial support in order to continue their outreach work within these communities.

Even though some CBOs have been operating in Vietnam since 2009, there has been, to our knowledge, no formal research on the working relationship between CBOs and government agencies. This information is urgently needed, because financial support from the Vietnamese government will be necessary for CBOs’ survival after international funding disappears. Therefore, this study aims to characterize CBOs’ outreach efforts and activities, past and present collaborations with Vietnamese government programs, and the barriers to and facilitators of such collaborations.

Based on the study findings, we will discuss recommendations aimed at improving collaborations between the CBOs and the government programs and strategies to promote the sustainability of CBO HIV/AIDS prevention and health promotion in communities of drug users and sex workers.

## Methods

### Study design

The paucity of research currently available and limited number of experts on the purpose of the CBOs, the interactions between the CBOs and the government programs, and health promotion in communities of drug users and sex workers led us to select an exploratory and qualitative research approach. We estimated that 24 interviews with peer educators (from either sex work or drug user CBOs) would ensure geographic representation from the three cities with CBOs (Hanoi, Ho Chi Minh City, and Hai Phong). Given the limited number of ministry officials and direct healthcare providers with experience working with CBOs, we believed that a total of eight interviews would be both feasible and adequate to identify some of the salient themes in the range of opinions on the questions we posed to these individuals. The Institutional Review Boards of the Yale University, USA, and the Institute for Social Development Studies, Vietnam, approved this study and all associated materials.

### Study participants

Eligible participants were at least 18 years of age and were working as drug use or sex work peer educators, government officials, or healthcare providers. Peer educators had to be employed by one of the CBOs supported by SCDI for at least 3 years, have good understanding about CBO activities, and be willing to be interviewed. These peer educators were selected as a discrete group from the larger group of outreach workers employed by the CBOs. Government officials were provincial DSEP staff, healthcare providers worked at clinics specializing in MMT or treatment of HIV/AIDS and sexually transmitted infections, needed to be currently engaged in direct or indirect collaboration with the CBOs, and expressed a willingness to be interviewed.

### Procedures

SCDI—a local non-governmental organization, legally registered under Vietnamese law—was the local host of the research. SCDI made logistic arrangements, nominated interviewees, arranged appointments for interviews, and obtained all necessary legal assurances that the fieldwork could be conducted. Using purposive sampling [18], all three categories of eligible participants—CBO peer educators, government officials, and healthcare providers—were recruited by SCDI staff. Prior to the interview, participants provided informed consent; peer educators consented verbally to protect their anonymity as members of vulnerable populations, while government officials and healthcare providers provided written consent. At the end of the interview, each participant received a gift value of 100,000 Vietnamese đồng (approximately US $5).

The lead author conducted all interviews in Vietnamese between June and August 2013. Each interview lasted approximately 50 minutes and was audio-recorded with the participants’ permission. All interviews were individual and face-to-face, with all but one occurring in private rooms or isolated spaces at either the participants’ or interviewer’s workplace, as these locations were convenient for and acceptable to participants. However, one government official in Hanoi preferred to meet in the back room of a café near his workplace; the reason for this preference was not indicated.

The Yale research team developed a preliminary version of the interview guide that was subsequently reviewed by the leadership at SCDI to ensure the relevance of the questions. The guide included a set of demographic items (i.e., sex, age, education level, job title, and years at current job), administered before the open-ended questions. The major topics explored during the interviews were (1) CBO activities and functions, (2) the history of CBO-government program collaboration(s), and (3) perceived barriers, facilitators, and willingness of participants to undertake CBO-government program collaboration.

Cooperation between the Yale and SCDI researchers included weekly meetings of the lead author and SCDI staff to discuss the ongoing recruitment and iterative data analytic process during the lead author’s stay in Vietnam. Two SCDI staff members transcribed verbatim the recorded interviews. Prior to transcribing, these two individuals signed a nondisclosure agreement to keep the content of the interviews confidential. The lead author proofread each transcript, anonymized the data, and created summaries of all interview transcripts in English to generally familiarize co-authors who did not understand Vietnamese. In addition, the lead author was in residence at SCDI for the entire summer, she and senior SCDI staff could immediately discuss research issues as they arose, and the entire research team continued to communicate regularly as data analysis and manuscript preparation continued once the lead author returned to the USA.

### Data analysis strategy

All interview transcripts were read and coded using thematic analysis [19]. An initial coding schema was developed based on the three initial project objectives: (1) to assess the functions of the CBOs, (2) to understand the history of the CBO-government program collaborations, and (3) to identify the barriers to and facilitators of the current working relationship between CBOs and government programs. The coding schema was subsequently modified during the iterative data analytic process; “negative” instances (i.e., comparative analysis that may not fit initial constructs) were sought in order to expand, adapt, or restrict the original conceptual scheme.

The lead author is fluent in both languages, Vietnamese being her first language. She was responsible for coding all of the Vietnamese language transcripts. To ensure that there was agreement about code definitions and how they were applied to the interviews, however, three transcripts were translated into English and coded independently by two members of the research team (LTL and LEG). Any differences in coding were discussed and resolved by clarifying definitions of codes or modifying codes to improve reliability. Two translated transcripts were coded at the beginning of the study and another midway through the study to prevent drift in the application of the codes.

For the analysis, all codings were conducted on the Vietnamese language transcripts. LTL and LEG met biweekly to discuss the data analysis, identify emergent themes, and introduce new codes as needed. All transcripts were coded in ATLAS.ti, version 7.0 (Scientific Software Development GmbH). Quotations to be used in any English language manuscript or report were translated by the lead author. All authors from SCDI and Yale reviewed and discussed the results and agreed on the study findings.

## Results

### Description of sample

Characteristics of the study sample appear in Table 1. In all three cities, drug use peer educators were divided—eight males and six females—while all sex work peer educators were female. The gender distribution among the government officials and healthcare providers was nearly evenly divided. The majority of peer educators had some secondary-level schooling (grades 9–12), while all government officials and healthcare providers interviewed in Hanoi and Ho Chi Minh City reported at least some college-level education. The age of the study participants ranged from 28 to 63 years, with a mean age of 33 years. Government officials and healthcare providers in Hai Phong were unavailable for interview during the summer of 2013.

**Table 1:** Demographic characteristics of the total interview sample (*N* = 32)

Total sample				
Characteristics	Drug use peer educators	Sex work peer educators	Government officials	Direct healthcare providers
	(*n* = 14)	(*n* = 10)	(*n* = 5)	(*n* = 3)
Region				
Hanoi	7	4	4	1
Ho Chi Minh City	4	3	1	2
Hai Phong	3	3	0	0
Sex				
Female	5	10	3	2
Male	8	0	2	1
Transgender	1	0	0	0
Education Level				
Primary school	1	2	0	0
Lower secondary school (7–8)	1	5	0	0
Secondary school (9–12)	10	3	0	0
Some college	2	0	5	3
Mean age	36.4	36.7	32.3	26.3
Mean years at current job^a^	3.2	3.2	5.0	5.3

^a^Some participants reported the number of years they have been in the types of work, rather than the number of year they have been in their current jobs

### Interview themes

**Fig. 1:**
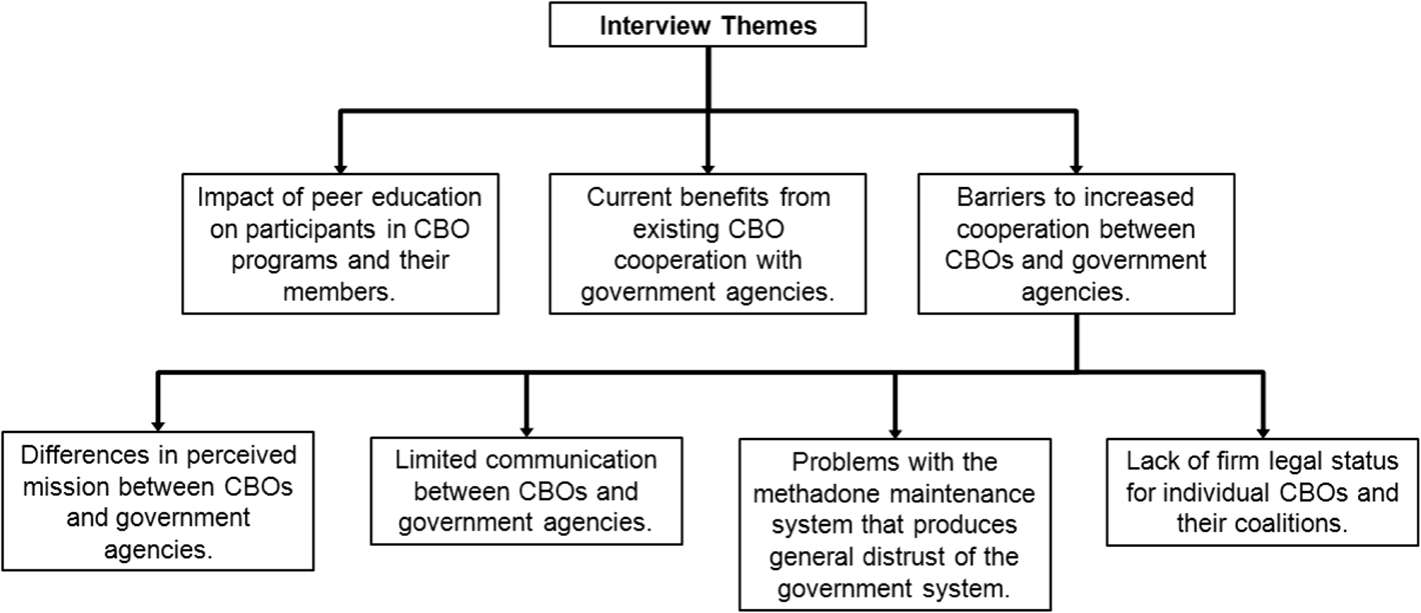
Themes and sub-themes identified from interviews with peer educators

The coding schema was designed to focus attention on the operations of peer educators and the barriers they face in trying to increase the benefits they can provide for the populations they serve. Three major themes were identified from the peer educator interviews: the impact of peer education on participants in CBO programs and their members, current benefits from existing CBO cooperation with government agencies, and barriers to increased cooperation. The last major theme included four subthemes: differences in perceived mission between CBOs and government agencies, limited communication between them, problems with the methadone maintenance system that produces general distrust of the government system, and lack of firm legal status for individual CBOs and their coalitions (Fig. 1).

The themes identified in the interviews with government officials and direct healthcare service providers were, by and large, consistent with the predominant opinions of the peer educators. Where substantial differences between the peer educators and the two other groups were identified, these are discussed in detail within the relevant subsection.

### CBOs’ impact on their members

The services provided by the CBOs were considered by peer educators, government officials, and healthcare providers to positively influence CBO members’ quality of life. This is consistent with data collected from participants in CBO programs in Hanoi [20]. All groups agreed that CBO members enjoyed multiple benefits, such as educational workshops, support for social interaction with family and neighbors, referral to healthcare services, and general socio-emotional support. Peer educators felt that their personal experience with drug use, sex work, or both enabled them to share information about safe practices in drug use and sex work to their members.

*“When the members get to share [their experiences] with each other at the CBOs, they become more confident about life.” (Female DSEP field staff member)*

*“Because I used to use drugs, I can see the difficulties of the drug users and the social stigma towards drug users.” (Male drug use peer educator)*

A similar perception was expressed by government officials:

*“The outreach workers help their members very effectively because they share similar experiences. There’s nothing like people with similar circumstances helping each other, understanding each other’s illnesses, perceptions, likes and dislikes.” (Male DSEP deputy director)*

Also, as clients of HIV-related services, the peer educators themselves have good knowledge of and connection to the service system. They described how they use this knowledge to benefit the CBO members they serve.

*“We have to connect with the clinics for voluntary testing for HIV and tuberculosis, so that our members don’t have to pay fees” (Male drug use peer educator)*

With the expansion in the availability of methadone as treatment for opioid addiction, CBOs can play a crucial role in referring active drug users to treatment, assisting with the entry process, and promoting adherence and retention in care.

*“…if [the members] haven’t gotten notification about methadone [quota for patient enrollment] so that they can go to the police department [to get their applications certified], then it will surely be very hard. But with the CBO’s counsel, [the members] will be more confident.” (Male drug use peer educator)*

Support for methadone maintenance was universal among the healthcare providers.

*“When methadone applicants participate at the CBOs, they reduce their drug use dosage. This is really good because these applicants will have an easier time quitting [drugs] completely once they enroll into [the MMT program].” (Female healthcare provider)*

Some drug user and sex worker CBOs have united to form coalitions that operated under a common coordinating board and within a shared office space. In Hanoi, the Coming Home Coalition further collaborated with a CBO of intimate partners of drug users; in Ho Chi Minh City, the Towards the Future Coalition collaborated with a CBO of men who have sex with men and a CBO of male sex workers. In contrast to the CBOs in Hanoi and Ho Chi Minh City, we were unaware of any current CBO coalition in Hai Phong, although the participating drug user and sex worker CBOs in Hai Phong had entered into a coalition that terminated in less than 1 year. We were unable to obtain information about the reasons for this dissolution.

One of the reported benefits of a coalition was increasing efficiency in meeting members’ specific service needs by permitting outreach workers to recommend members to another CBO within the same coalition.

*“Here we have [members] who are 2 in 1 [or] 3 in 1, that is, they are at the same time drug users, people living with HIV, and sex workers—three things that need support. So when [we] established a coalition, we were able to easily refer them.” (Male drug use peer educator)*

*“It’s good that [CBOs in a coalition] can support each other. For example, the [members] in the sex worker CBO who don’t use drugs will have intimate partners who do. Then there are some drug users with intimate partners who are sex workers. The wife can participate with our sex worker CBO, while the husband can participate with our drug user CBO.” (Female sex work peer educator)*

Another reported benefit of a coalition was the opportunity for capacity building among outreach workers. Operating within a shared office space, outreach workers from different CBOs were able to learn various outreach skills from peer educators and from each other, enabling them to more effectively interact with potential members with multiple vulnerabilities.

### Mutual benefits from CBO-government program collaborations

Collaboration between the CBOs and the local government officials was mutually beneficial while also serving CBO members’ needs. DSEP field staff at the provincial level interacted more often with local CBOs than did more senior government employees (e.g., local deputy directors). They visited the CBO offices to monitor operations, learn about CBO operational models, update CBO staff about relevant changes in the legal system, schedule visits to the detention centers, solicit feedback about local events and their potential implications for relevant policy development, and serve as liaisons between CBOs and the more senior government officials.

*“I introduce [outreach workers] to the [detention] centers. Actually it’s from an assignment by my superior. [The CBOs] have to submit proposals to DSEP. Then DSEP delegates me or another associate to respond to [the CBOs] and notify the centers. I monitor the [outreach workers’] activities and remind them of the centers’ internal policies. [Outreach workers] can ask me if they need consultation. [If] regarding laws, then I can come teach them about it.” (Male DSEP field staff member)*

A less formal type of collaboration involved the local government authorities giving verbal permission for the CBOs to operate in the community.

*“We have to send monthly reports of our plans and operations to the ward. Other than that, the local police department gives us certificates recognizing our contributions to the community. But there isn’t a single document permitting this group to work here.” (Female sex work peer educator)*

CBOs and the local government-run MMT clinics collaborated to help the clinics meet their quotas for patient enrollment. The collaboration, in turn, benefited CBOs and their members by reducing potential barriers to entry into the MMT program.

*“[The CBOs] are good sources for [methadone] patient referrals because the [drug users] trust the [outreach workers] more. If [drug users] are afraid to come out, the [outreach workers] will assure them that the clinic doctors will keep their drug use status confidential. And they will be more willing to come to this clinic.” (Female healthcare provider)*

Collaborations between CBOs and government programs sometimes resulted in providing CBO members with financial support. At the time of the study, the DSEP provincial offices in Hanoi and Ho Chi Minh City offered vocational training programs to teach eligible sex workers, aged 18–24 years, basic skills in hairdressing, barbering, manicuring, and applying make-up. Officials contacted local CBOs to request referrals of sex worker members to participate in the 3-month training, which, in Hanoi, also provided a stipend of 730,000 Vietnamese đồng (approximately US $34.60) per month for commuting purposes.

### Barriers and challenges preventing improved CBO-government program collaborations

Barriers emerged in four areas: (1) perceived conflicting missions of the CBOs and the government organizations in addressing drug use and sex work in the community, (2) limited communications, (3) CBO mistrust of the MMT system, and (4) lack of legal status for CBOs.

#### Perceived differences in missions

CBOs aimed to reduce the harmful effects of drug use and sex work, whereas the local police wanted to eradicate these activities. Peer educators reported that many outreach workers were reluctant to be seen interacting with the local police officials when doing street outreach as potential CBO members might then distrust them.

*“I know some of the [local policemen], but I don’t dare interact with them. It’s a very sensitive issue. There will be problems if the [drug user members] see me talking with [the policemen]. The [drug user members] might hurt me. So I just avoid the [policemen] altogether.” (Male drug use peer educator)*

*“I don’t know what to say if I go to the [government officials]. Their job is to beautify the city and keep it clear [of bad influences]. I want to reach out to the sex worker sisters. How could [the officials] accept our work?” (Female sex work peer educator)*

#### Limited communication hampers cooperation

Government officials recognized their lack of direct interaction with members of these marginalized communities. This caused them to be seen as out of touch by lower ranking government officials and CBO outreach workers, as well as prevented the establishment of meaningful working relationships or the sharing of resources with local CBOs.

*“Those [officials] at higher ranking may only be sitting behind their desks, or they’ve perhaps heard [about the CBOs] through reports. They rarely interact with these [outreach workers]. I am 100 % confident that they have a different outlook.” (Female DSEP field staff member)*

#### CBO mistrust of the methadone treatment system

Barriers and challenges to collaborations between the participating CBOs and several local MMT clinics included a need for bribes to gain entrance into some clinics and the stringent application requirements. This theme largely relied upon data from the peer educator group as very few healthcare providers were interviewed. A drug use peer educator noted she and some of her outreach workers learned that some members had been required to “pay [healthcare personnel] tens of millions of [Vietnamese] đồng^1^ to [receive MMT].” This practice among some healthcare personnel prevented the CBOs from forming collaborative relationships with local MMT clinics known for corrupt practices.

*“[Applicants] have to pay money to get in the queue [for enrollment into the MMT program]. When [the applicants] lose that money, they will immediately think that [the CBO] benefited from the [payment]. Therefore, [my CBO] doesn’t want anything to do with that methadone program.” (Male drug use peer educator)*

Additionally, the application for entry into MMT programs required disclosure of drug use status. Hence, potential applicants for the treatment program risked being sent to the 06 centers.

*“We have to fight for those [drug users] who have all the qualifications to get [methadone] treatment so they won’t get sent away [to the detention centers].” (Male drug use peer educator)*

#### CBOs need legal status to become legitimate organizations

When the government formally recognizes a given Vietnamese organization, the organization is then officially granted legal status and is then officially able to participate in government programs and personally represent themselves when applying for and receiving financial support from domestic and international philanthropic organizations.

Currently, most CBOs rely upon non-governmental organizations (e.g., SCDI, the Hanoi and Ho Chi Minh City HIV/AIDS Associations, or non-governmental organizations that implement Global Fund-supported projects) to apply for and accept funding from donors. Hence, obtaining legal status is crucial to CBO autonomy and long-term sustainability.

*“[Legal status] is required in administrative work. They [need to] have a stamp to confirm their positions, [to be] recognized by the government. So when these CBOs want to work with other organizations, they have to show their position in society. Who are [the CBOs]? Where are they from? Who provided the paperwork for their activities? It’s to show that [they are] grounded.” (Male DSEP field staff member)*

A widely held belief among peer educators was that gaining legal status would also foster social acceptance within the Vietnamese society. As one noted, “If our role is recognized, then the stigmatization in the community towards [sex work] would reduce.”

During the time of the study, no policies existed for defining the qualifications CBOs needed in order to achieve legal status. Therefore, the CBOs were presented with a challenge from the government officials who spoke of the need for them to publicly demonstrate their role in benefiting society as a prerequisite for obtaining legal status. By contrast, the peer educators claimed to not know exactly the criteria by which their CBOs can attain legal status.

*“I think that we do need [legal status], but the problem [is] there are no places for us to [apply] for it.” (Female sex work peer educator)*

## Discussions and recommendations

The testimonies shared by the participating drug use and sex work peer educators, government officials, and healthcare providers indicated a potential for CBOs and government programs to complement each other’s work. However, these entities must first overcome the existing barriers to collaboration between CBOs and government programs, to entry into the MMT program, and to obtaining legal status for the CBOs.

CBO outreach workers have access to the marginalized populations in Vietnam in ways unavailable to the relevant government officials and healthcare providers. Peer educators report that CBO staff have become a known and trusted source of information for drugs users and sex workers and can serve as a bridge between the target populations and existing government programs. Hence, they can effectively encourage members to avail themselves of services such as MMT, general healthcare, and vocational training.

Based on our analysis, we recommend that government programs increase collaborations with CBOs to effectively reach drug user and sex worker communities, reduce health risks, and improve access to services (Table 2). One strategy that is now being actively discussed is for healthcare centers to provide outreach workers with stations within the healthcare facilities where they can answer questions and guide the drug user and sex worker patients through the healthcare process. Currently, the outreach workers are only able to refer the drug user and sex worker members to healthcare centers. However, with the ability to guide the members through the medical care process, the outreach workers would be better able to ensure that the members obtain the needed services and adhere to treatment [21, 22].

**Table 2:** Recommendations to increase benefits of CBOs serving marginalized populations

Major themes	Specific recommendations
Increase collaboration with government agencies	(1) Station outreach workers in government healthcare centers
	(2) Develop case management skills among peer educators
Decrease barriers to methadone maintenance	(1) Expand availability from current 16,000 patients towards the 200,000 needed
	(2) Prevent “payment for access” schemes that create financial barriers to entry
Ensure continued funding and sustainability	(1) Obtain direct government funding for specific health promotion activities
	(2) Raise funds from non-governmental sources through grants and research collaborations
	(3) Train leadership at each CBO in grant writing, organizational management, network building
Achieve full government recognition	(1) Develop a clear statement of standards needed for recognition
	(2) Continue CBO coalition building to be able to speak with one voice to government agencies

A challenge to the CBOs’ increased collaboration with government programs is the potential loss of trust by their drug user or sex worker members who may fear that outreach workers, working in the clinics, will report the names of the members to local law enforcers. Therefore, CBOs need to help their members understand the changing nature of the CBO-government program collaboration and allay suspicions the members may have about the CBOs colluding with the government’s attempt to use the criminal justice system to restrict the liberty of CBO members.

Derived from our analysis of reports by the peer educators, the issue of bribery limits access into the MMT program for many eligible applicants. Bribery practices at several local methadone clinics may be motivated by the limited slots available within these MMT programs. Over 200,000 people in Vietnam are identified as PWID, but there are only 80 MMT clinics in 30, mainly major, provinces; these serve less than 16,000 patients [1, 23]. After the pilot MMT program ended in 2009, the Vietnamese government set a new goal to establish up to 245 MMT clinics in 30 provinces throughout Vietnam by the year 2015 to treat up to 80,000 opioid users [24]. Although this fivefold expansion is necessary, the government also needs to ensure that their program is fully accessible to all opioid users throughout Vietnam. Otherwise, methadone maintenance treatment will remain as limited as before and as prone to abuse. A first step for the government in making MMT programs fully accessible to drug users is to commission a taskforce of drug use and sex work outreach workers that would officially enable them to recruit in areas surrounding the MMT clinics. The legitimized role and acting in cooperation with the government program can potentially reduce barriers posed by local law enforcement officials who might not understand the roles of the outreach workers and simultaneously empower outreach workers in each geographical region.

To our knowledge, there has not been any research or report on the nature of corruption within the MMT program in Vietnam. Perhaps, it is because this issue is still relatively new since the treatment program was first piloted in 2008. There is a need for further research on the functioning of Vietnamese MMT clinics to understand the sources of corruption and to determine if expansion of the MMT clinics can reduce the prevalence of bribery.

More broadly, for the CBOs to remain a viable resource within Vietnam, they will need continued funding. Along with building their capacity to operate and serve the community, CBOs will need to obtain legal status to counteract the diminishing support from non-governmental organizations, and so they can represent themselves when submitting proposals and accepting grants from donors. Legal status will provide the opportunity for CBOs to speak directly with officials and civil society groups in order to advocate for and reduce stigma towards marginalized groups.

There was no clear consensus among the peer educators and government officials about the requirements for CBOs to qualify for legal status. This lack of a clear policy regarding application for legal status threatens the ability for the CBOs to prepare adequately for the withdrawal of international donors and to independently solicit sponsorship within Vietnam. Therefore, a clear and consistent set of standards for obtaining legal status for all CBOs should be established. One approach to establishing set standards is for the CBOs and their representative non-governmental organizations to provide government agencies with annual reports that document CBO achievements and capabilities. Over time, the accumulation of data on the CBOs’ operations, management, and accomplishments will potentially be sufficient evidence for government agencies to establish standards for applying for legal status.

The findings in this study point to the urgent need for CBOs to obtain legal status in order to ensure their sustainability. However, although legal status is a necessary condition, it may not be sufficient to ensure the CBOs’ ability to successfully attain sponsorship. Strong skills in grant writing and network building, along with effective planning, managing, and evaluation of CBO functioning, are among the essential components that will sustain the CBOs over time [25]. This may be an area for future studies to investigate and develop the “complete survival package” tailored to CBOs serving marginalized groups in Vietnam.

### Limitations of the study

Several limitations of the study should be noted. Since there were not enough direct healthcare providers with more than a superficial perspective on the benefits versus negative consequences of MMT, we were unable to reach saturation for this category of participants, especially regarding the dynamics between the CBOs and local MMT clinics. Additionally, as a result of the sampling strategy, the study reflects the perspectives of only those CBOs, government officials, and healthcare providers with whom SCDI has a working relationship. Further research in other cities and among organizations and individuals not affiliated with SCDI would be necessary to determine whether the current findings apply to other contexts [26]. Nonetheless, the qualitative data were systematically collected across a comprehensive set of key informants, settings, and times [27]. This approach to collecting and analyzing qualitative data permitted us to draw the following conclusion with some certainty that they represented the dominant opinion of the group of individuals we interviewed, while also considering negative and contradictory opinions [28]. The findings represent a rich, nuanced, and complex understanding of key stakeholders’ perceptions and attitudes concerning CBOs and should be taken into consideration when suggesting ways to improve collaborations between CBOs and local government programs.

## Conclusions

Despite considerable reform in Vietnam’s response to drug use and sex work, further improvements to reduce barriers that seem to restrict the CBO-government program collaborations are needed. Improvements might include consultative services at government healthcare centers, CBO outreach to promote access to a scaled-up MMT program, and establishment of clearly specified standards by which CBOs can obtain legal status. We hope our findings and recommendations will promote ongoing dialog among key players in the field that ultimately yield public health benefits in Vietnam.

## Abbreviations

CBO: Community-based organizations
MMT: Methadone maintenance treatment
HIV: Human immunodeficiency virus
AIDS: Acquired immunodeficiency syndrome
PWID: People who inject drugs
DSEP: Department of Social Evils Prevention
MoLISA: Ministry of Labor, War Invalids, and Social Affairs
SCDI: Center for Supporting Community Development Initiatives

## Footnotes

1 10 million đồng is approximately US $450. In 2012, the median monthly income is reported to be US $185 (http://www.thanhniennews.com/business/vietnam-average-monthly-wage-rises-to-185-8544.html).
